# Azilsartan, an angiotensin II type 1 receptor blocker, restores endothelial function by reducing vascular inflammation and by increasing the phosphorylation ratio Ser^1177^/Thr^497^ of endothelial nitric oxide synthase in diabetic mice

**DOI:** 10.1186/1475-2840-13-30

**Published:** 2014-01-31

**Authors:** Sachiko Matsumoto, Michio Shimabukuro, Daiju Fukuda, Takeshi Soeki, Ken Yamakawa, Hiroaki Masuzaki, Masataka Sata

**Affiliations:** 1 Department of Cardiovascular Medicine, The University of Tokushima Graduate School of Health Biosciences, 3-18-15 Kuramoto, Tokushima 770-8503, Japan; 2 Department of Cardio-Diabetes Medicine, The University of Tokushima Graduate School of Health Biosciences, 3-18-15 Kuramoto, Tokushima 770-8503, Japan; 3 Division of Endocrinology, Diabetes and Metabolism, Hematology, Rheumatology (Second Department of Internal Medicine), Graduate School of Medicine, University of the Ryukyus, Okinawa, Japan

## Abstract

**Background:**

Azilsartan, an angiotensin II type 1 (AT1) receptor blocker (ARB), has a higher affinity for and slower dissociation from AT1 receptors and shows stronger inverse agonism compared to other ARBs. Possible benefits of azilsartan in diabetic vascular dysfunction have not been established.

**Methods:**

We measured vascular reactivity of aortic rings in male KKAy diabetic mice treated with vehicle, 0.005% azilsartan, or 0.005% candesartan cilexetil for 3 weeks. Expression of markers of inflammation and oxidative stress was measured using semiquantitative RT-PCR in the vascular wall, perivascular fat, and skeletal muscle. Phosphorylation of endothelial nitric oxide synthase (eNOS) at Ser^1177^ and Thr^495^ was measured using Western blotting, and the ratio of phosphorylation at Ser^1177^ to phosphorylation at Thr^495^ was used as a putative indicator of vascular eNOS activity.

**Results:**

(1) Vascular endothelium–dependent relaxation with acetylcholine in KKAy mice was improved by azilsartan treatment compared to candesartan cilexetil; (2) the ratio of Ser^1177^/Thr^495^ phosphorylation of eNOS was impaired in KKAy and was effectively restored by azilsartan; (3) anomalies in the expression levels of monocyte chemotactic protein 1 (MCP1), F4/80, NAD(P)H oxidase (Nox) 2, and Nox4 of the aortic wall and in the expression of TNFα in the perivascular fat were strongly attenuated by azilsartan compared to candesartan cilexetil.

**Conclusions:**

These results provide evidence that azilsartan prevents endothelial dysfunction in diabetic mice, more potently than does candesartan cilexetil. Azilsartan’s higher affinity for and slower dissociation from AT1 receptors may underlie its efficacy in diabetic vascular dysfunction via a dual effect on uncoupled eNOS and on Nox.

## Introduction

In patients with type 2 diabetes mellitus (T2DM), both macrovascular and microvascular disease cause extensive morbidity and mortality [1,2]. Treatment with angiotensin-converting enzyme inhibitors [3] and angiotensin II type 1 (AT1) receptor blockers (ARBs) [4] improves both macrovascular and microvascular outcomes in patients with T2DM. The renin–angiotensin system (RAS), a hormonal cascade that includes angiotensinogen, renin, angiotensin-converting enzyme, angiotensin, and its receptors is involved in the maintenance of systemic blood pressure. Alternatively, angiotensin II functions as a local biologically active mediator in the progression of cardiovascular remodeling through the AT1 receptor [5]. Therefore, ARBs are thought to have cardioprotective effects beyond their antihypertensive effects. In a diabetic state, excessive systemic production of angiotensin II or predominant intracrine or intracellular RAS activation might be involved in the progression of vascular complications [6,7]. Therefore, elucidating effects and mechanisms of action of ARBs is crucial for understanding diabetic vascular complications.

Endothelial nitric oxide synthase (eNOS) is a nitric oxide synthase that generates nitric oxide (NO) in blood vessels and is involved with regulating vascular tone by inhibiting smooth muscle contraction [8]. Loss of NO bioavailability is believed to indicate a dysfunctional phenotype across broad properties of the endothelium. Thus, the assessment of its vasodilator properties resulting from NO can provide information on the integrity and function of the endothelium. Such endothelial dysfunction is implicated in the pathogenesis of cardiovascular diseases of type 2 diabetes [9].

Enzymatic activity of eNOS is regulated by multiple phosphorylation of specific sites on the eNOS protein [10]. The most well-studied are the functional consequences of phosphorylation of Ser^1177^ and Thr^495^. Ser^1177^ is a positive regulatory site of eNOS, and Thr^495^ is a negative regulatory site of eNOS in that phosphorylation leads to increase or decreased enzymatic activity [10]. It has been reported that drugs interfering with the renin-angiotensin–aldosterone system enhances eNOS phosphorylation at Ser^1177^ and improves NO bioavailability [11,12]. However, these beneficial effects of RAS blockade are not inspected in diabetic models. Reactive oxidant species (ROS), which are produced at a high rate in the diabetic and/or insulin resistant obese state [13], can cause oxidative damage of cellular components and activate several pathways linked with inflammation. RAS have been identified in different organs, most notably in those playing a significant role in metabolism and insulin sensitivity, including the liver, skeletal muscle and adipose and perivascular tissue. It has been reported that inhibition of RAS reduce ROS production pathways such as Nox2, a major catalytic component of an endothelial NADPH oxidase [14], and Nox4, a component of endothelial and smooth muscle NADPH oxidase [15], proinflammatory markers such as tumor necrosis factor α (TNFα) [16], monocyte chemotactic protein 1 (MCP1) [16], F4/80 (a marker for mature macrophages and monocytes) [17] and improve adipocyte-expression of peroxisome proliferator–activated receptor γ_2_ (PPARγ_2_), the ligand-activated nuclear hormone receptor [18], insulin receptor substrate 1 (IRS-1) [19], and adiponectin [20].

A new ARB, azilsartan, was recently approved and is expected to exert a more potent, sustained for 24 h BP-lowering effect compared to existing ARBs (candesartan cilexetil, olmesartan, telmisartan, valsartan, and irbesartan) [21]. In an *in vitro* study, it has been shown that azilsartan has higher affinity for and slower dissociation from AT1 receptors [22] and shows stronger inverse agonism [23]. These effects of azilsartan on the AT1 receptor may underlie its superior BP-lowering properties (compared to other ARBs) and may be beneficial in diabetic vascular remodeling.

The present study was designed to compare the efficacy of azilsartan and candesartan cilexetil against abnormalities in vascular reactivity and eNOS phosphorylation (which reflects eNOS inactivation [24-26]) and against ROS and inflammatory activation in the vascular wall, perivascular fat, and skeletal muscle in a murine diabetic model.

## Materials and methods

### Animals and the protocol

Eight-week-old male KKAy mice (Clea Japan Inc., Tokyo, Japan) were randomly distributed into 3 treatment groups so that the groups had similar average blood glucose level and body weight. The mice were fed a standard moderate-fat (MF) diet or an MF diet mixed with 0.005% candesartan cilexetil or 0.005% azilsartan (Takeda Pharmaceutical Co., Ltd., Osaka, Japan) for 3 weeks (KKAy-Vehicle, KKAy-Azilsartan, and KKAy-Candesartan groups). Age-matched C57BL/6 J male mice (SLC Japan) were used as a control group (C57BL/6 J-Vehicle group). Based on a report showing that two-week treatment with candesartan cilexetil had a cardioprotective effects in 8-week-old KKAy mice [27], we chose a 3-week treatment period in 8-week old KKAy mice. Body weight, food intake, and systolic blood pressure were checked weekly. Blood pressure was measured using a tail cuff system (Softron, Co., Tokyo). At the end of the experimental period, we collected plasma, subcutaneous fat, visceral fat, liver, pancreas, soleus muscle, quadriceps muscle, thoracic aorta, and perivascular fat around the thoracic aorta and stored them at -80°C until analysis. Blood glucose levels were measured by a glucose meter, plasma insulin levels by a mouse insulin ELISA kit (Shibayagi Co.,Ltd., Gunma, Japan) and uric acid by a uric acid assay kit (Cayman Chemical, Ann Arbor, USA). Mice were housed in a light- and temperature-controlled room in a 12-hour light/dark cycle. All animal experiments were approved by the Committee on Animal Research, the University of Tokushima and have been conducted in accordance with international ethical principles and guidelines for experiments on animals.

### The glucose tolerance test and insulin tolerance test

After 2 weeks of treatment, glucose tolerance test (GTT) and insulin tolerance test (ITT) were performed. In GTT, the mice were given an intraperitoneal injection of 1 gram of glucose per kilogram of body weight after 16 hours of overnight fasting. Blood glucose levels were measured in 0, 15, 30, 60, 90, and 120 min. In ITT, the mice were given an injection of 0.75 or 1 unit of insulin per kilogram of body weight after 4 hours of fasting on post-GTT Day 3. Insulin sensitivity was estimated by percent changes in the plasma glucose concentration.

### Vascular reactivity

The analysis of vascular reactivity was performed as described previously [28]. Briefly, the periadventitial tissue of the descending thoracic aorta was dissected away under a stereomicroscope into the Krebs–Henseleit buffer (KHB; 118.4 mM NaCl, 4.7 mM KCl, 2.5 mM CaCl_2_, 1.2 mM KH_2_PO_4_, 1.2 mM MgSO_4_, 25 mM NaHCO_3_, 11.1 mM glucose) and cut transversely into ~2.0-mm-long ring segments. The aortic rings were placed in a tissue chamber filled with KHB (37°C) bubbled with 90% O_2_–5% CO_2_ and were mounted onto 2 tungsten wires for measurement of isometric tension. Initial tension was set at 0.7 g, and the rings were allowed to equilibrate as the chambers were refilled with fresh KHB every 15 min. Cumulative concentration–response curves for acetylcholine (Ach; 10^-9^ M to 10^-4^ M) and sodium nitroprusside (SNP; 10^-9^ M to 10^-4^ M) were generated after induction of an approximately 60% contraction with phenylephrine (Phe) of maximal contraction caused by 31.4 mM KCl.

### Western blotting analysis

For immunoblotting, we homogenized tissues in RIPA buffer (Wako Pure Chemical Industries, Ltd., Tokyo) containing protease inhibitors (Takara Bio Inc., Shiga, Japan) and a phosphatase inhibitor (Nacalai Tesque, Kyoto, Japan) and collected the supernatants. Proteins (5 μg/lane) were separated on 5–20% gradient SDS–polyacrylamide gels and transferred onto polyvinylidene difluoride membranes (GE Healthcare Bio Sciences, Piscataway, NJ). After blocking with TBS-T buffer containing either 3% bovine serum albumin or 5% skim milk, the membranes were incubated with antibodies against phospho-eNOS (Ser^1177^, 1:1000, Cell Signaling Technology, Beverly, MA), phospho-eNOS (Thr^495^, 1:1000, Cell Signaling Technology) and total eNOS (1:1000, BD Biosciences, San Diego, CA) or a peroxidase-conjugated antibody against β-actin (1:50000, Sigma–Aldrich, St. Louis, MO) [29] and antibody binding was detected with horseradish peroxidase-conjugated secondary antibodies (1:2000; Chemicon) using an enhanced chemiluminescence system (GE Healthcare Japan, Tokyo). The band intensity of phospho-eNOS (Ser^1177^ and Thr^495^) was scanned in gray scale at the maximum resolution of at least 600 dpi using NIH Image J 1.47 and arbitrary ratio normalized to the band intensity of total-eNOS were used.

### Semiquantitative RT-PCR analysis

After extraction of total RNA from aorta, perivascular fat, and soleus muscle, we synthesized cDNA, using the QuantiTect Reverse Transcription kit (Qiagen, Valencia, CA) and then, performed real-time RT-PCR with gene-specific primers and SYBR green dye on an Applied Biosystems 7500 Real-Time PCR System (Life Technologies Japan Ltd, Tokyo). The forward (fwd) and reverse (rev) primer sequences are as follows: TNFα [30] (fwd: 5′-ACCCTCACACTCAGATCATCTTC-3′; rev: 5′-TGGTGGTTTGCTACGACGT-3′), MCP1 [30] (fwd: 5′-CCACTCACCTGCTGCTACTCAT-3′; rev: 5′-TGGTGATCCTCTTGTAGCTCTCC-3′), F4/80 [17] (fwd: 5′-TGCATCTAGCAATGGACAGC-3′; rev: 5′-GCCTTCTGGATCCATTTGAA -3′), Nox2 [14] (fwd: 5′-ACTCCTTGGGTCAGCACTGG-3′; rev: 5′-GTTCCTGTCCAGTTGTCTTGG-3′), Nox4 [15] (fwd: 5′-TGTTGGGCCTAGGATTGTGTT-3′; rev: 5′-AGGGACCTTCTGTGATCCTCG-3′), adiponectin (fwd: 5′-ATGGCAGAGATGGCACTCCT-3′; rev: 5′-CCTTCAGCTCCTGTCATTCCA-3′), PPARγ_2 _[18] (fwd: 5′-GARGGAAGACCACTCGCATT-3′; rev: 5′-AACCATTGGGTCAGCTCTTG -3′), IRS-1 [19] (fwd: 5′-GCCAGAGGATCGTCAATAGC-3′; rev: 5′-AAGACGTGAGGTCCTGGTTG-3′), and β-actin (fwd: 5′-CCTGAGCGCAAGTACTCTGTGT-3′; rev: 5′-GTCGATCCACATCTGCTGGAA-3′).

### Statistical analysis

Data were calculated as mean ± SEM. All analyses were performed using the Prism software (version 6.0d, GraphPad Software, La Jolla, CA, USA). Two-way analysis of variance was used for the changes of systolic blood pressure and body weight in the course of administration, and for GTT and ITT. Differences between multiple groups were analyzed by one-way analysis of variance or the Kruskal–Wallis test in the case of non-Gaussian distribution, followed by the Tukey's *post hoc* test for comparison between treatment groups. Differences with p < 0.05 were considered statistically significant and with p < 0.01 significant.

## Results

### General characteristics

**Figure 1:**
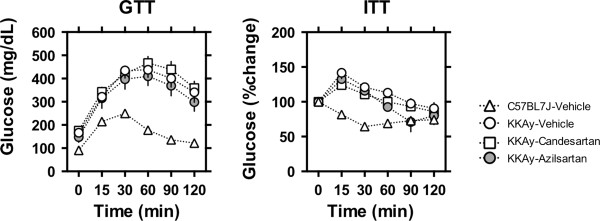
**Glucose tolerance test (GTT) and insulin tolerance test (ITT).** GTT and ITT were performed in male KKAy mice fed standard moderate fat (MF) diet (KKAy-Vehicle, ○) and MF mixed with 0.005% candesartan cilexetil (KKAy-Candesartan groups, □) or 0.005% azilsartan (KKAy-Azilsartan, ●) for 2 weeks since 8–9 weeks of age. Age-matched C57BL/6 J male mice were used as a control group (△). The data are shown as mean ± SEM.

As shown in Table 1, body weight increased to the same extent in KKAy mice treated with vehicle, candesartan cilexetil, or azilsartan, and blood pressure was reduced comparably by candesartan cilexetil and azilsartan. In GTT, blood glucose levels in KKAy mice were higher at baseline and were strongly elevated after a glucose load compared to age-matched C57BL/6 J (Figure 1). In ITT, blood glucose levels did not decrease after an insulin injection in KKAy mice. Treatment with either candesartan cilexetil or azilsartan in KKAy mice did not change the response curves in GTT and ITT. Plasma levels of uric acid increased comparably in KKAy mice treated with vehicle, candesartan cilexetil, or azilsartan as compared to C57BL/6 J (Table 1). Weight of subcutaneous and visceral fat and of the liver was elevated and weight of the soleus and quadriceps muscles decreased in KKAy mice. Those metrics were not changed by candesartan cilexetil and azilsartan treatment (Table 1).

**Table 1:** General characteristics

			**C57BL6J-Vehicle**			**KKAy-Vehicle**				**KKAy-Candesartan**					**KKAy-Azilsartan**		
Number				7			9				9				8		
Body weight																	
	baseline	(g)	22.4	±	0.4	36.5	±	1.3		37.2	±	1.6		37.0	±	1.6	
	1 week	(g)	23.9	±	0.6	41.2	±	0.8	††	40.8	±	0.9	††	41.3	±	0.9	††
	2 week	(g)	24.3	±	0.6	40.5	±	0.7	††	40.5	±	0.9	††	40.6	±	0.9	††
	3 week	(g)	25.1	±	0.8	43.8	±	0.6	††	43.1	±	0.9	††	43.0	±	0.8	††
Systolic blood pressure																	
	baseline	(mmHg)	101	±	1	101	±	3		100	±	2		102	±	2	
	1 week	(mmHg)	100	±	2	109	±	2	†	82	±	3	**	84	±	3	**
	2 week	(mmHg)	102	±	1	104	±	2		81	±	2	**	82	±	2	**
	3 week	(mmHg)	99	±	2	108	±	2	†	79	±	2	**	82	±	3	**
Fasting blood glucose																	
	baseline	(mg/dL)	53	±	4	75	±	21		78	±	8		77	±	7	
	2 week	(mg/dL)	63	±	4	139	±	22	†	104	±	10	†	138	±	14	†
Plasma uric acid																	
	3 week	(μmol/L)	6.5	±	1.8	18.7	±	1.5	†	18.4	±	3.0	†	18.0	±	2.5	†
Tissue weight (g/g)																	
subcutaneous fat/body weight			0.015	±	0.002	0.040	±	0.001	††	0.038	±	0.002	††	0.042	±	0.001	††
visceral fat/body weight			0.014	±	0.001	0.025	±	0.001	††	0.024	±	0.001	††	0.028	±	0.001	††
liver/body weight			0.047	±	0.002	0.064	±	0.002	††	0.058	±	0.002	††	0.066	±	0.001	††
soleus muscle/body weight			0.013	±	0.000	0.008	±	0.000	††	0.009	±	0.000	††	0.007	±	0.000	††
quadriceps muscle/body weight			0.014	±	0.001	0.009	±	0.000	††	0.010	±	0.000	††	0.009	±	0.000	††

Values are mean ± SEM. **p<0.01 vs KKAy-Vehicle. † p<0.05 and †† p<0.01 vs C57BL6J-Vehicle.

### Effects on vascular function in KKAy mice

**Figure 2:**
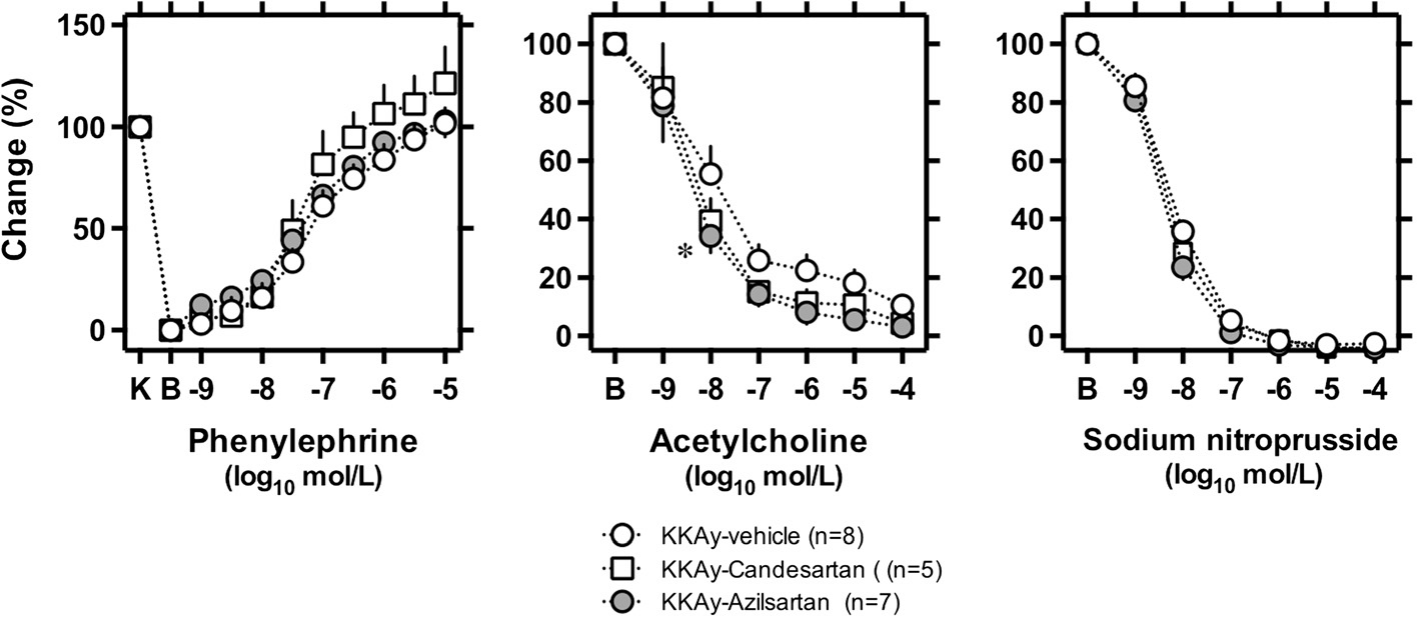
**Vascular reactivity in aorta ring.** Vascular reactivity to phenylephrine, acetylcholine, or sodium nitroprusside was determined using aortic rings isolated from male KKAy mice fed standard moderate fat (MF) diet (KKAy-Vehicle, ○) and MF mixed with 0.005% candesartan cilexetil (KKAy-Candesartan groups, □) or 0.005% azilsartan (KKAy-Azilsartan, ●) for 3 weeks since 8–10 weeks of age. Data represent the mean ± SEM. *p < 0.05, KKAy-vehicle versus KKAy-Azilsartan.

As shown in Figure 2, vascular endothelium–dependent relaxation in response to acetylcholine in KKAy mice was improved significantly by azilsartan, but not by candesartan cilexetil. The half-maximal effective concentration (EC50) for acetylcholine was -8.06 ± 0.16 (log_10_ mol/L) in vehicle-treated KKAy, -8.31 ± 0.16 in candesartan-treated KKAy (p = 0.120 compared to vehicle), and -8.41 ± 0.14 in azilsartan-treated KKAy (p = 0.005 compared to vehicle). Constriction in response to phenylephrine and endothelium-independent relaxation in response to sodium nitroprusside did not differ among vehicle-, candesartan cilexetil–, and azilsartan-treated KKAy mice.

### Effects on total eNOS and eNOS phosphorylation at Ser^1177^ and Thr^495^

**Figure 3:**
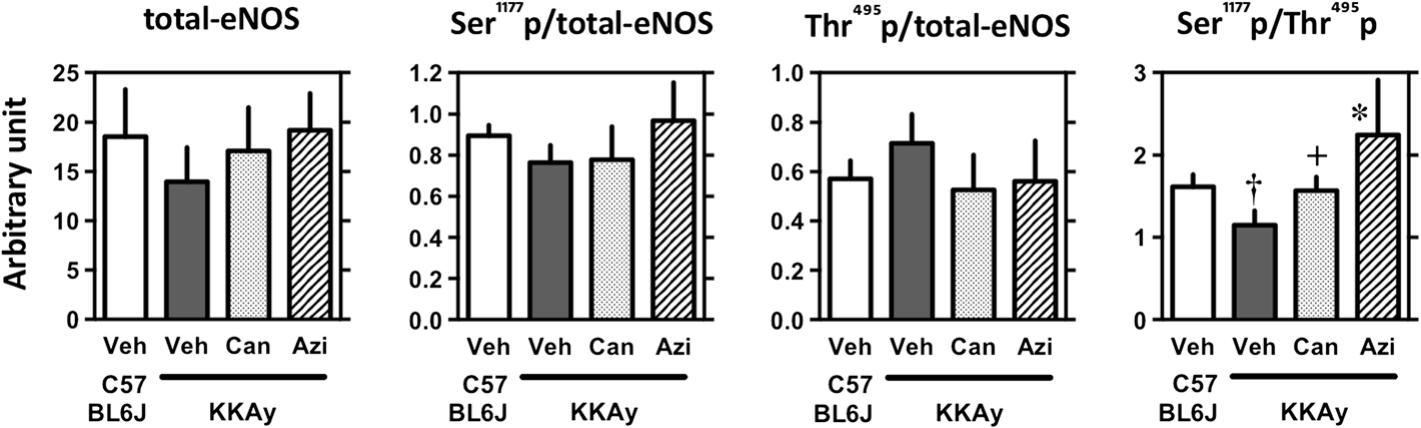
**Relative intensity of total eNOS and eNOS phosphorylation at Ser**^**1177 **^**(Ser**^**1177**^**p) and Thr**^**495 **^**(Thr**^**495**^**p) and the ratio of eNOS phosphorylation at Ser**^**1177 **^**to at Thr**^**495 **^**(Ser**^**1177**^**p/Thr**^**495**^**p; lower panel) in aorta.** Using proteins (5 μg/lane) of aorta isolated from male KKAy mice fed standard moderate fat (MF) diet (Veh) and MF mixed with 0.005% candesartan cilexetil (Can) or 0.005% azilsartan (Azi) for 3 weeks since 8–10 weeks of age, Western blotting analysis were made. Age-matched C57BL/6 J male mice were used as a control group. After transfer, the membranes were incubated with antibodies against phospho-eNOS (Ser^1177^, 1:1000, Cell Signaling Technology, Beverly, MA), phospho-eNOS (Thr^495^, 1:1000, Cell Signaling Technology) and total eNOS (1:1000, BD Biosciences, San Diego, CA) and antibody binding was detected with horseradish peroxidase-conjugated secondary antibodies (1:2000; Chemicon) using an enhanced chemiluminescence system (GE Healthcare Japan, Tokyo). The band intensity of phospho-eNOS (Ser^1177^ and Thr^495^) was scanned in gray scale at the maximum resolution of at least 600 dpi using NIH Image J 1.47 and arbitrary ratio normalized to the band intensity of total-eNOS were used. Data represent mean ± SEM. ^†^p < 0.05 compared to C57BL/6 J and ^+^p < 0.1 and *p < 0.05 compared to KKAy-vehicle, according to the Kruskal–Wallis test.

Phosphorylation at Ser^1177^, a positive regulatory site of eNOS associated with increased enzymatic activity [10], tended to be decreased, and phosphorylation at Thr^495^, a negative regulatory site of eNOS associated with decreased enzymatic activity [10], tended to be increased in KKAy mice compared to control mice (C57BL/6 J). Although changes in phosphorylation signals either at Ser^1177^ or Thr^495^ did not reach statistical significance, the ratio of phosphorylation at Ser^1177^ to phosphorylation at Thr^495^ was statistically significantly lower in KKAy mice (Figure 3). Compared to candesartan cilexetil, azilsartan was effective at restoring the phosphorylation ratio Ser^1177^/Thr^495^ (p < 0.05 compared to KKAy-vehicle).

### Effects on gene expression in aorta, perivascular fat, and soleus muscle

**Figure 4:**
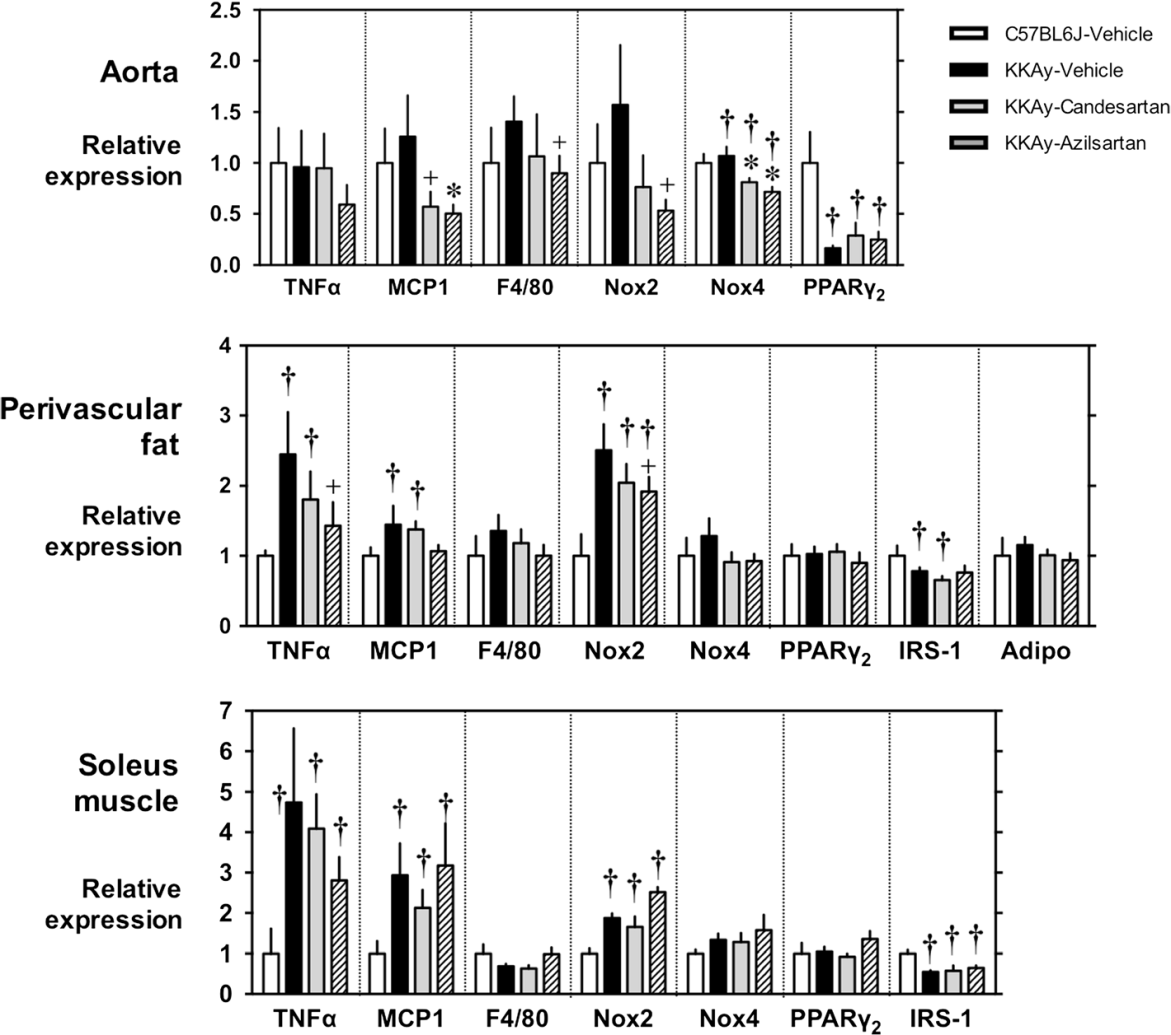
**Gene expression in aorta (upper panel), perivascular fat (middle panel), and soleus muscle (lower panel).** Samples isolated from male KKAy mice fed standard moderate-fat (MF) diet (black bars) and MF diet mixed with 0.005% candesartan cilexetil (gray bars) or 0.005% azilsartan (shaded bars) for 3 weeks since 8–10 weeks of age, were analyzed by semiquantitative RT-PCR. *Gapdh* was used as an internal control. Values shown represent mean ± SEM (n = 4–6).^†^p < 0.05 compared to C57BL/6 J and ^+^p < 0.1 and *p < 0.05 compared to KKAy-vehicle according to either the Kruskal–Wallis test or unpaired *t* test. TNFα: tumor necrosis factor α; MCP1: monocyte chemotactic protein 1; PPARγ: peroxisome proliferator–activated receptor γ_2_; IRS-1: insulin receptor substrate 1; and Adipo: adiponectin.

In the aortic wall of KKAy mice, mRNA expression of MCP1, a representative inflammatory cytokine [16], of F4/80, a marker for mature macrophages and monocytes [17], of Nox2, a major catalytic component of an endothelial NADPH oxidase [14], and of Nox4, a component of endothelial and smooth muscle NADPH oxidase [15], tended to be increased in KKAy mice compared to control mice (C57BL/6 J; Figure 4, upper panel). These levels of mRNA expression were strongly reduced by azilsartan compared to candesartan cilexetil. Expression of PPARγ_2_, the ligand-activated nuclear hormone receptor [18], was decreased in KKAy, but was not altered by either candesartan cilexetil or azilsartan. In the perivascular fat, mRNA expression of TNFα, MCP1, and Nox2 was increased in KKAy mice (Figure 4, middle panel), and these anomalies in the expression of TNFα and Nox2 were attenuated only by azilsartan. In the soleus muscle of KKAy, mRNA expression levels of TNFα, MCP1, and NOX2 were increased and that of IRS-1 [19] was decreased (p < 0.05 compared to the control group C57BL6J-Vehicle), and these anomalies of KKAy mice were not attenuated by either candesartan cilexetil or azilsartan (Figure 4, lower panel).

## Discussion

The major findings of the present study are as follows: (1) vascular endothelium–dependent relaxation in response to acetylcholine in KKAy mice was improved strongly by azilsartan compared to candesartan cilexetil; (2) the ratio of Ser^1177^/Thr^495^ phosphorylation of eNOS, a putative marker for eNOS activation was impaired in KKAy mice, and the healthy ratio was effectively restored by azilsartan compared to candesartan cilexetil; (3) the differences in the expression levels of MCP1, F4/80, Nox2, and Nox4 of the aortic wall and in the expression of TNFα in the perivascular fat were attenuated by azilsartan compared to candesartan cilexetil. These data suggest that azilsartan restores endothelial function more effectively than does candesartan cilexetil, by normalizing eNOS function and by reducing inflammation and oxidative stress in diabetic mice.

Vascular endothelial dysfunction is implicated in the pathogenesis of cardiovascular diseases [24] and is well known to occur in obesity [31] and type 2 diabetes [9]. The present study compared the efficacy of azilsartan and candesartan cilexetil on vascular reactivity, which reflects eNOS inactivation [24-26], and we measured phosphorylation of vascular eNOS as an indicator of eNOS activity. Enzymatic activity of eNOS is regulated by multiple phosphorylation of specific sites on the eNOS protein [10]. When Ser^1177^ is phosphorylated, nitric oxide production is increased 2- to 3-fold. In contrast, Thr^495^ is a negative regulatory site of eNOS in that phosphorylation leads to decreased enzymatic activity [10]. It has been reported that phosphorylation of Ser^1177^-eNOS is decreased in diabetic rats [32] and diabetic patients [33]. Inversely, eNOS phosphorylation at Ser^495^ was reported to be increased in *db/db* diabetic mice [34]. These data are consistent with our results showing that diabetic KKAy mice have a tendency for lower phosphorylation at Ser^1177^ and stronger phosphorylation at Thr^495^; these mice show a significantly decreased phosphorylation ratio of Ser^1177^ to Thr^495^ on eNOS compared to control C57BL6J mice. The Ser^1177^/Thr^495^ ratio was effectively normalized (increased) by azilsartan treatment compared to candesartan cilexetil. The kinases AMPK, Akt, protein kinase A (PKA), calmodulin/Ca^2+^ dependent protein kinase (CaMKII), protein kinase G (PKG) and the phosphatase protein phosphatase 2A (PP2A) have all been implicated in the regulation of eNOS-Ser^1177^ phosphorylation [10], while protein kinase C (PKC) has been shown to phosphorylate eNOS-Thr^495^. In addition, there is evidence for co-ordination between dephosphorylation of eNOS-Thr^495^ and activating phosphorylation of eNOS-Ser^1177 ^[35]. The mechanism(s) for the alteration of phosphorylation at Ser^1177^ and Thr^495^ in the diabetic state are largely unknown, however, altered signaling in AMPK, Akt, PKA, CaMKII, PKG, PP2A, and PKC in the diabetic state could be involved in the deregulated eNOS phosphorylation.

In the aortic wall of KKAy mice, mRNA expression levels of MCP1 and F4/80 tended to be increased, suggestive of overproduction of proinflammatory cytokines by activated macrophages/monocytes. Such proinflammatory cytokines frequently cause overproduction of ROS via excessive stimulation of reduced nicotinamide adenine dinucleotide phosphate [36]. Cellular sources of ROS include NADPH-dependent oxidases, xanthine oxidase, lipoxygenases, mitochondrial oxidases, and NO synthases [37]. NADPH oxidase (Nox) is a major source of ROS in diabetic humans [38] and diabetic animals [39]. Seven isoforms of Nox have been described in mammals [36]. Each isoform contains a core catalytic subunit, i.e., Nox1–Nox5 and dual oxidase (DUOX) 1 and DUOX 2 [36]. Each Nox catalytic isoform contains up to 5 regulatory subunits that determine 1) maturation and expression of Nox and DUOX subunits in biological membranes, 2) enzyme activation, and 3) spatial organization. In our study, mRNA expression levels of Nox2 (gp91phox), the major catalytic component of endothelial NADPH oxidase, and Nox4, a component of endothelial and smooth muscle NADPH oxidase [36], were increased, suggesting that ROS production is increased via overexpression of Nox2 and Nox4 in KKAy mice. Reportedly, a siRNA-mediated knockdown of Nox2 (which is upregulated in diabetic endothelial cells) reduces ROS production and improves vascular function [14]. It has also been reported that Nox4 is upregulated in an animal diabetic model [40] or as a result of hyperglycemia [41], with a concomitant increase in ROS. Taken together, upregulation of Nox2 and/or Nox4 in the aorta may be linked to ROS overproduction and vascular dysfunction in our murine model of diabetes.

The upregulation of ROS-producing Nox2 and Nox4 was decreased strongly by azilsartan as compared to candesartan cilexetil, according to the present results. We could not determine whether the greater inhibition of aortic Nox2 and Nox4 expression by azilsartan significantly sensitizes endothelial vasodilator response to acetylcholine. Angiotensin II levels are increased in patients with diabetes [42] and hyperglycemia potently upregulated expression of the angiotensin II type 1 receptor (AT1) [43]; thus, both could sensitize vascular cells to angiotensin II. Oak and Cai reported that streptozotocin-induced diabetes in mice is characterized by a marked increase in aortic ROS production, which is inhibited by NG-nitro-L-arginine methyl ester hydrochloride (L-NAME, inhibitor of nitric oxide synthase) in contrast to nondiabetic controls, indicating uncoupling of eNOS in the diabetic state [44]. According to their data, angiotensin II receptor type 1 blocker candesartan decreased eNOS-derived ROS while augmenting nitric oxide bioavailability in diabetic aortas, which is suggestive of recoupling of eNOS. Nox activity was more than doubled in the endothelium-denuded diabetic aortas but this effect was attenuated by candesartan, indicating that Nox remains active in nonendothelial vascular tissues, although uncoupled eNOS is responsible for endothelial production of O_2_. They concluded that the dual effect on uncoupled eNOS and Nox might explain the high efficacy of angiotensin II antagonists in restoring endothelial function [44].

Going back to our results, because azilsartan has higher affinity for and slower dissociation from AT1 receptors [22] and shows stronger inverse agonism [23], these effects of azilsartan on AT1 receptor, as compared with candesartan cilexetil, may underlie the superior efficacy in diabetic vascular dysfunction via the dual effect on uncoupled eNOS and Nox. There is a report showing that angiotensin II-induced contraction was augmented in aorta rings isolated from diabetic rats and suggesting that the enhanced functional coupling of AT1 receptors results in supersensitivity to Ang II [45]. The higher affinity of azilsartan for AT1 receptors may be beneficial for protecting angiotensin II-induced vascular remodeling in the diabetic condition [42]. Clinical studies have exhibited that some benefits conferred by ARBs may not be class effects, but rather molecular effects [46]. It was shown in a clinical study that in losartan users uric acid levels decrease from baseline, while they increase in users of other ARBs like valsartan, telmisartan, candesartan, and olmesartan [47]. Among these ARBs, losartan uniquely exhibits a cis-inhibitory effect on the uptake of uric acid by the renal uric acid transporter (URAT1) [48]. Partial chemical structures for the URAT1 competitive binding may involve an AT1 receptor-independent mechanism of action [48]. In our study, plasma levels of uric acid increased comparably in KKAy mice treated with vehicle, candesartan cilexetil, or azilsartan as compared to C57BL/6 J, indicating no difference in uric acid metabolism between two ARBs. It has been reported that genetic disruption or pharmacological inhibition by telmisartan of the AT1R attenuates atherosclerosis and improves endothelial function in diabetic ApoE-/- mice via the PPARγ pathway [49]. In 3 T3-L1 preadipocytes, azilsartan enhanced adipogenesis as well as effects on expression of PPARα, PPARδ, leptin, adipsin, and adiponectin [50]. Azilsartan also potently inhibited vascular cell proliferation in the absence of exogenously supplemented angiotensin II or in cells lacking AT1 receptors [50]. These findings suggest that azilsartan can function as a pleiotropic ARB with beneficial effects on actions that could involve more than just blockade of AT1 receptors and/or beyond their antihypertensive effects.

In the perivascular fat, mRNA expression levels of TNFα, MCP1, and Nox2 were increased in KKAy, and the overexpression of TNFα and Nox2 was attenuated only by azilsartan. Aortic expression of PPARγ_2_ was decreased in KKAy, but was not altered by candesartan cilexetil and azilsartan. We previously demonstrated that adiponectin secreted from perivascular adipose tissue has a protective role in neointimal formation after endovascular injury thanks to its anti-inflammatory properties, whereas perivascular adipose tissue–secreted TNFα plays an adverse atherogenic role in neointimal formation because of its proinflammatory effects [51,52]. Kurata et al. reported that blockade of angiotensin II receptor ameliorates adipocytokine dysregulation and that such action is mediated, at least in part, by a reduction of oxidative stress in accumulated adipose tissue [20]. In agreement with the previous report, mRNA expression of TNFα, MCP1, and Nox2 was increased in the perivascular fat from KKAy mice, and these anomalies in the expression of TNFα and Nox2 were attenuated only by azilsartan. Although the current study could not verify the direct link between vascular dysfunction and attenuation of adipocytokine dysregulation in perivascular fat, the possible role of perivascular fat in azilsartan-induced vascular remodeling should be assessed in future studies.

Although weight in the liver was increased, weight in the soleus and quadriceps muscle was decreased in the KKAy mice. A relative decrease of muscle mass as compared to body weight fails to metabolize abundant fat and worsens fat accumulation in the liver. Abundant fat with concomitant metabolic derangement underlies vascular dysfunction in the KKAy diabetic mice [53,54]. In the soleus muscle of KKAy, mRNA expression levels of TNFα, MCP1, and Nox2 were increased and IRS-1 was decreased, but this overexpression was not attenuated by either candesartan cilexetil or azilsartan. It has been shown that azilsartan reduced left ventricular hypertrophy, cardiac fibrosis, plasminogen activator inhibitor-1 (PAI-1; a marker of profibrosis) in aortic banding mice fed high-fat diet [55], indicating that azilsartan may exert favorable biological effects in non-diabetic obese insulin-resistant condition, which shares a common mechanism such as enhanced ROS/inflammation signals with the current model.

## Conclusions

Our data suggest that azilsartan restores endothelial function more effectively than does candesartan cilexetil, by normalizing eNOS function and by reducing inflammation and oxidative stress in diabetic mice.

## Abbreviations

AT1: Angiotensin II type 1 (AT1) receptor; ROS: Reactive oxygen species; NAD(P)H: Nicotinamide adenine dinucleotide phosphate; Nox: NAD(P)H oxidase; eNOS: Endothelial nitric oxide synthase; L-NAME: *N*^G^-nitro-L-arginine methyl ester; TNFα: Tumor necrosis factor-α; MCP1: Monocyte chemotactic protein 1; PPARγ: Peroxisome proliferator–activated receptor-γ; IRS-1: Insulin receptor substrate 1.

## Competing interests

The authors declare that they have no competing interests.

## Authors’ contributions

SM designed and performed study, MiS designed the study and wrote the manuscript, DF and TS were involved in discussions, KY and HM participated in vascular function study, and MaS designed and supervised this study. All authors read and approved the final manuscript.
